# The rapidly advancing Class 2 CRISPR‐Cas technologies: A customizable toolbox for molecular manipulations

**DOI:** 10.1111/jcmm.15039

**Published:** 2020-02-10

**Authors:** Jingyi Wang, Chenzi Zhang, Bo Feng

**Affiliations:** ^1^ Key Laboratory for Regenerative Medicine, Ministry of Education School of Biomedical Sciences, Faculty of Medicine CUHK-GIBH Joint Research Laboratory on Stem Cells and Regenerative Medicine The Chinese University of Hong Kong Hong Kong SAR China; ^2^ Institute for Tissue Engineering and Regenerative Medicine (iTERM) The Chinese University of Hong Kong Hong Kong SAR China; ^3^ Guangzhou Institute of Biomedicine and Health, Chinese Academy of Sciences Guangzhou Regenerative Medicine and Health Guangdong Laboratory Guangzhou China

**Keywords:** base editor, CRISPR imaging, CRISPR‐based genome‐wide screening, CRISPR‐Cas systems, gene edting, genome editing, RNA editing

## Abstract

The CRISPR‐Cas technologies derived from bacterial and archaeal adaptive immune systems have emerged as a series of groundbreaking nucleic acid‐guided gene editing tools, ultimately standing out among several engineered nucleases because of their high efficiency, sequence‐specific targeting, ease of programming and versatility. Facilitated by the advancement across multiple disciplines such as bioinformatics, structural biology and high‐throughput sequencing, the discoveries and engineering of various innovative CRISPR‐Cas systems are rapidly expanding the CRISPR toolbox. This is revolutionizing not only genome editing but also various other types of nucleic acid‐guided manipulations such as transcriptional control and genomic imaging. Meanwhile, the adaptation of various CRISPR strategies in multiple settings has realized numerous previously non‐existing applications, ranging from the introduction of sophisticated approaches in basic research to impactful agricultural and therapeutic applications. Here, we summarize the recent advances of CRISPR technologies and strategies, as well as their impactful applications.

## 1 INTRODUCTION

The clustered regularly interspaced short palindromic repeats (CRISPR)‐CRISPR‐associated (Cas) systems are naturally occurring adaptive immune systems in many bacteria and archaea, combating invading viruses and plasmids.^1^ They act in three steps to provide protection from foreign invaders: (a) adaptation, during which a series of Cas proteins mediate the acquisition of invading nucleic acid fragments (spacers) into the CRISPR array loci of host cells; (b) biogenesis, which includes constitutive transcription from the CRISPR array followed by maturation of CRISPR RNA (crRNA) and continuous expression of Cas protein(s); and (c) targeting (also called interference), in which a crRNA would guide the effector complex containing Cas nuclease(s) to cleave homologous sequence(s) to destroy the invading nucleic acids.^2^ The mechanism underlying the targeting step was found of great value to mediate RNA‐guided DNA cleavage and has been exploited for programmable genome targeting. Compared to earlier tools developed for genome editing, such as zinc‐finger nucleases (ZFNs) and transcription activator‐like effector nucleases (TALENs), which rely on protein‐DNA interactions for targeting and can only be programmed through protein engineering to modify their DNA‐binding domains to re‐target different sequences, the CRISPR‐Cas systems provide much greater ease for reliable reprogramming.^3^ The specificity of targeting is conferred by RNA‐based guidance through base‐pairing, and the guide sequences can be easily adjusted to target a new sequence with high certainty.^3^ Therefore, this breakthrough has actualized the long‐desired site‐specific genome editing with high efficiency, high accuracy and ease of reprogramming.

According to the number of proteins involved in the targeting step, CRISPR‐Cas systems are generally classified into two classes named Class 1 and Class 2.^4^ In Class 1 systems, multiple protein units form an effector complex together with the crRNA to recognize and cleave a target sequence, whereas a single protein complexing with crRNA does the job in a Class 2 system.^4^ To date, there are six types of CRISPR‐Cas systems discovered: three of them (type I, type III and type IV) are identified as Class 1 systems, while the other three (type II, type V and type VI) are classified into the Class 2 category.^4^ Due to the straightforward composition of the effectors, Class 2 systems have been intensively studied, engineered and applied for genome editing, and among which, the type II (Cas9) systems are the most thoroughly characterized and utilized.^5^, ^6^

**Figure 1:**
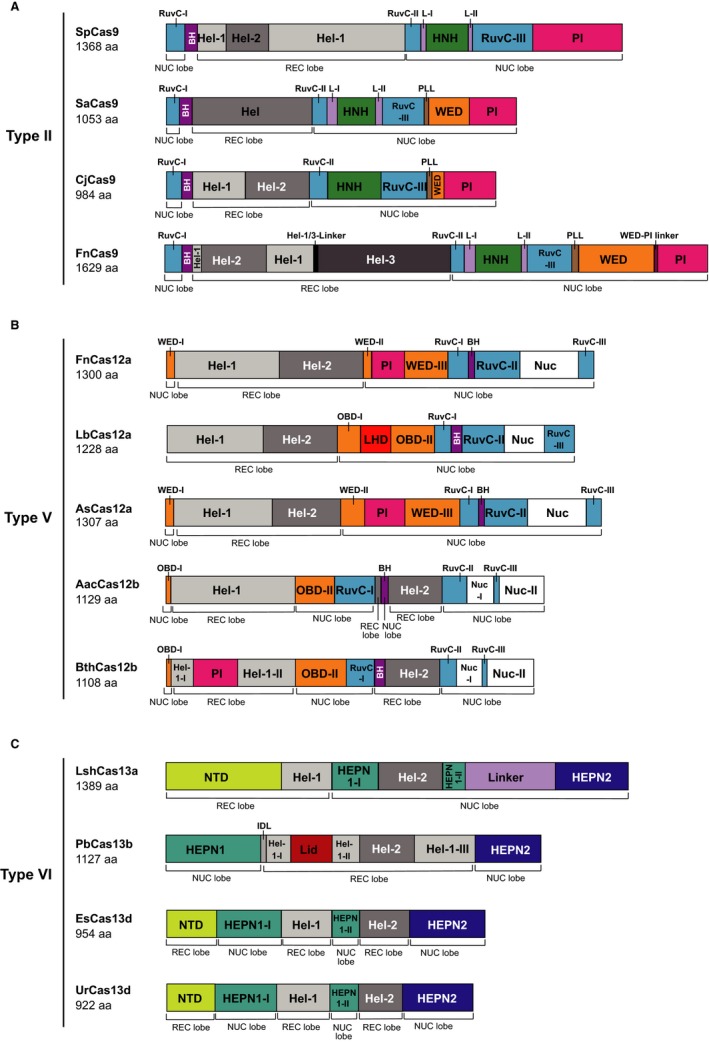
Schematic representation of among Class 2 CRISPR‐Cas effectors whose crystal structures have been determined. A, The domain organizations of type II effectors, SpCas9,^8^, ^18^ SaCas9,^8^, ^20^ CjCas9,^21^ and FnCas9.^8^, ^30^ B, The domain organizations of type V effectors, FnCas12a,^140^ LbCas12a,^38^ AsCas12a,^39^ AacCas12b,^33^, ^44^ and BthCas12b.^141^ C, The domain organizations of type VI effectors, LshCas13a,^50^ PbCas13b,^142^ EsCas13d,^52^ and UrCas13d.^53^ BH, bridge helix; Hel, helical; IDL, inter‐domain linker; L, linker; LHD, looped‐out helical domain; NTD, N‐terminal domain; OBD, oligonucleotide‐binding domain; PI, PAM‐interacting; PLL, phosphate lock loop; WED, wedge. OBD and WED are equivalent nomenclature for the same domain

In 2013, the Cas9 system was first applied in mammalian cells for site‐specific genome editing, and the success has greatly encouraged investigations regarding other CRISPR‐Cas systems for potentially better editing efficiency and novel applications.^5^, ^6^ Not only have additional type II systems been discovered from new species, but also new members of type V and type VI systems are being identified and characterized (Table 1 and Figure 1).^7^ Along with the delineation of detailed structures and acting mechanisms of various Class 2 CRISPR‐Cas systems,^4^, ^7^, ^8^ optimizations and new applications have been conceived and accomplished. In this article, we will review representative members of Class 2 CRISPR‐Cas systems that have been discovered so far, and discuss their wide‐ranging applications in gene‐editing, novel tools developed therefrom, as well as some prospective advancement of the CRISPR‐Cas technology.

**Table 1:** Effectors of characterized Class 2 CRISPR‐Cas systems

Type	Effector	Biological origin	Modification when characterized	Size (aa)	Nuclease domain(s)	Spacer	tracrRNA	PAM/PFS^e^	Cleaving target	DSB	Refs
II	SpCas9	*Streptococcus pyogenes*	D10A/C80L/C574E/H840A	1368	RuvC & HNH	20 nt	Yes	5'‐(PS)‐NGG	dsDNA	Blunt	18
II	SaCas9	*Staphylococcus aureus*	N580A/C946A	1053	RuvC & HNH	20 nt	Yes	5'‐(PS)‐NNGRRT	dsDNA	Blunt	20
II	CjCas9	*Campylobacter jejuni*	—	984^b^	RuvC & HNH	22 nt	Yes	5'‐(PS)‐NNNNRYAC	dsDNA; ssRNA	Blunt	21, 143
II	St1Cas9	*Streptococcus thermophilus*	—	1122	RuvC & HNH	20 nt	Yes	5'‐(PS‐)NNAGAAW	dsDNA	Blunt	144
II	NmCas9	*Neisseria meningitidis*	—	1083^b^	RuvC & HNH	24 nt	Yes	5'‐(PS)‐NNNNGATT	dsDNA	Blunt	27
II	FnCas9	*Francisella novicida*	—	1629	RuvC & HNH	21 nt	Yes	5'‐(PS)‐NGG	dsDNA; ssRNA	Blunt	30
II	RHA FnCas9	*Francisella novicida*	E1369R/E1449H/R1556A	1629	RuvC & HNH	21 nt	Yes	5'‐(PS)‐YG	dsDNA	Blunt	30
V‐A	FnCas12a (Cpf1)	*Francisella novicida*	—	1300	RuvC	23‐25 nt	No	5' (T)TTV‐(PS)	dsDNA; ssDNA	5' staggered	32, 41, 145
V‐A	LbCas12a (Cpf1)	*Lachnospiraceae bacterium*	—	1228	RuvC	23‐25 nt	No	5′ TTTV‐(PS)	dsDNA; ssDNA	5' staggered	38, 41, 146
V‐A	AsCas12a (Cpf1)	*Acidaminococcus *sp.	—	1307	RuvC	24 nt	No	5′ TTTN‐(PS)	dsDNA; ssDNA	5' staggered	39, 41, 146
V‐B	AacCas12b (C2c1)	*Alicyclobacillus acidoterrestris*	—	1129	RuvC	20 nt	Yes	5′ TTN‐(PS)	dsDNA; ssDNA	5' staggered	33, 34, 41, 44
V‐B	BthCas12b (C2c1)	*Bacillus thermoamylovorans*	—	1108	RuvC	19 nt	Yes	5′ ATTN‐(PS)	dsDNA	5' staggered	34
V‐B	AaCas12b (C2c1)	*Alicyclobacillus acidiphilus*	—	1129 b	RuvC	20 nt	Yes	5′ TTN‐(PS)	dsDNA	5' staggered	34
V‐B	BhCas12b v4	*Bacillus hisashii*	K846R/S893R/E837G	1108	RuvC	22 nt	Yes	5′ ATTN‐(PS)	dsDNA	5' staggered	147
V‐C	OspCas12c (C2c3)	*Oleiphilus *sp.	—	1252^c^	RuvC	—	Yes	5′ TG‐(PS)	dsDNA; ssDNA	—	35
V‐C1	Cas12c1 (C2c3)^a^	—	—	1302 c	RuvC	—	Yes	5′ TG‐(PS)	dsDNA; ssDNA	—	35
V‐C2	Cas12c2 (C2c3) a	—	—	1218 c	RuvC	—	Yes	5′ TN‐(PS)	dsDNA; ssDNA	—	35
V‐D	Cas12d (CasY) a	—	—	~1200	RuvC	17‐19 nt	No	5′ TA‐(PS)	dsDNA	—	36
V‐E	Cas12e (CasX) a	—	—	~980	RuvC	20 nt	Yes	5′ TTCN‐(PS)	dsDNA	—	36
V‐F	Cas14a1 a	—	—	500	RuvC	20 nt	Yes	5′ TTTR‐(PS)	dsDNA; ssDNA	5' staggered	37, 148
V‐G1	Cas12g1 a	—	—	767 c	RuvC	—	Yes	No PAM requirement	dsDNA; ssDNA	Not applicable	35
V‐H	Cas12h1 a	—	—	870 c	RuvC	—	No	5′ RTR‐(PS)	dsDNA; ssDNA	—	35
V‐I	Cas12i1 a	—	—	1093 c	RuvC	—	No‐	5′ TTN‐(PS)	dsDNA; ssDNA	—	35
VI‐A	LshCas13a (C2c2)	*Leptotrichia shahii*	—	1389	Helical‐1; 2 HEPN	14‐28 nt	No	5'‐(PS)‐H	ssRNA	Not applicable	45, 50
VI‐A	LwaCas13a (C2c2)	*Leptotrichia wadei*	—	1152 b	2 HEPN	28 nt	No	No PFS requirement	ssRNA	Not applicable	49
VI‐B1	BzCas13b (C2c6)	*Bergeyella zoohelcum*	—	1224 d	2 HEPN	30 nt	No	5′ D‐(PS)‐NAN/NNA	ssRNA	Not applicable	47
VI‐B2	PbCas13b (C2c6)	*Prevotella buccae*	—	1127 d	2 HEPN; Lid	30 nt	No	Similar to BzCas13b	ssRNA	Not applicable	47, 142
VI‐C	Cas13c (C2c7)	*Fusobacterium perfoetens*	—	1121	2 HEPN	—	—	—	ssRNA (predicted)	Not applicable	7
VI‐D	EsCas13d	*Eubacterium siraeum*	—	954	2 HEPN	22 nt	No	No PFS requirement	ssRNA	Not applicable	48, 52
VI‐D	UrCas13d	*Uncultured Ruminococcus *sp.	—	922 c	2 HEPN	20 nt	No	No PFS requirement	ssRNA	Not applicable	48, 52, 53

a Discovered from Metagenomic data.

b Codon‐optimized for human cells.

c Codon‐optimized for *E coli*.

d Codon‐optimized for mammalian cells.

e PS = protospacer; N = any base; R = A/G; Y = C/T; W = A/T; V = A/C/G; H = A/C/T; D = A/G/T.

## 2 TYPE II: THE CRISPR‐CAS9 SYSTEMS

The implementation of CRISPR‐Cas9 systems for genome engineering should acknowledge the research efforts from different fields that have been continued through more than 30 years.^9^ CRISPR sequences were first described to be arranged as direct repeats with spacers in 1987,^10^ after which the natural functions and working mechanisms were unveiled step by step, setting up the stage for the first piece of experimental evidence regarding CRISPR‐Cas‐mediated adaptive immunity in 2007.^11^ In 2013, marked by the first application of codon‐optimized Cas9 originated from *Streptococcus pyogenes*, type II systems became the first CRISPR‐Cas systems applied as programmable genome editing tools targeting mammalian cells,^5^, ^6^, ^12^ prompting extensive investigations into the phylogeny, functions and structural details of known and newly identified CRISPR‐Cas systems.

The type II CRISPR‐Cas systems operate through an effector module consisting of a single Cas9 protein, a crRNA and a trans‐activating crRNA (tracrRNA).^6^ Each crRNA carries a guide sequence (20 nt) derived from a spacer at its 5′ end, which is capable of base‐pairing with the homologous sequence found in the invader (target) DNA, and a repeat‐derived sequence of 19–22 nt in length at its 3′ end, which hybridizes with a tracrRNA via complementary sequence.^13^ The crRNAs are encoded by the CRISPR array in the host genome, which is first transcribed into a long pre‐crRNA composed of multiple crRNA sequences. The pre‐crRNA would then pair up with multiple tracrRNAs, each base‐paring with one of the repeat sequences, and be processed by RNase III into separate crRNA‐tracrRNA structures. Each crRNA, with its 3′ repeat sequence paired with a tracrRNA, would eventually form a complex with a Cas9 protein and act as a guide to aid the identification of any homologous sequences (target sites) found in the invader DNA.^13^, ^14^ These molecular principles have been exploited for targeting desired sequences through modifying the crRNA coding genes, thereby developing a programmable genome editing tool. Furthermore, scientists have successfully engineered a single RNA chimera that mimics the structure of tracrRNA:crRNA complex.^15^ This engineered single guide RNA (sgRNA) can fully replace the tracrRNA:crRNA to direct the Cas9 complex for sequence‐specific DNA cleavage, thus lowering the complexity of genome editing technology to a greater extent and providing the most commonly used CRISPR‐Cas9 system for genome editing nowadays.^15^

The base‐pairing process between a Cas9‐crRNA complex and its target sequence (also named protospacer) requires an additional short sequence located 3′ downstream to the target sequence on the non‐targeted strand, which is known as the protospacer‐adjacent motif (PAM).^16^ When Cas9 protein(s) in a complex recognizes a PAM sequence through its PAM‐interacting (PI) domain (Figure 1), it triggers a DNA melting process at the adjacent regions as well as subsequent base‐pairing between the crRNA (or sgRNA) and target sequence(s).^17^ The proper interaction between Cas9 protein(s) and PAM sequence(s) is often essential, and it significantly affects the efficiency and specificity of subsequent targeting and cleavage.^17^ The various Cas9 systems discovered from different species recognize distinct PAM sequences, which are effector‐specific and often G‐rich.^18^ For practical applications in genome editing, the requirement of PAM enhances the specificity while restricting the selection of target sites from a specified genome. Moreover, it is often found that several nucleotides (the “seed” sequence) in the guide sequence would be first exposed to the solvent environment, which facilitates the subsequent full base‐pairing process.^19^ Altogether, the PAM sequence and the “seed” sequence are critical for the specificity of Cas9‐catalysed DNA cleavage.^18^

Structural studies of representative Cas9 effectors reveal that most of them have a bi‐lobed architecture consisting of a recognition (REC) lobe and a nuclease (NUC) lobe (Figure 1A), which forms a positively charged groove to accommodate the negatively charged sgRNA:target DNA heteroduplex.^18^, ^20^, ^21^ The NUC lobe consists of three domains: the PI domain interacting with PAM, as well as the RuvC and HNH domains that cleave the non‐targeted strand and the target strand, respectively.^18^ After being guided to a target sequence by a specified sgRNA, both the RuvC and HNH domains in Cas9 catalyse DNA cleavage to introduce a double‐stranded break (DSB) with blunt ends.^18^

### 2.1 SpCas9

The *Streptococcus pyogenes* Cas9 (SpCas9) system mentioned above is the first and most widely applied CRISPR‐Cas system harnessed for genome editing.^5^, ^6^ SpCas9 protein has a size of 1368 amino acids (aa) (~4.1 kb), and it recognizes 5′ NGG as the PAM sequence (Table 1).^18^ The engineered SpCas9 system has been extensively applied in genome editing for various research and therapeutic purposes, from which two major concerns have been raised. Firstly, SpCas9 tolerates mismatches of up to several bases between the guide and target sequences, which could potentially induce off‐target mutagenesis in host cells.^22^, ^23^ Secondly, the SpCas9 protein is relatively large: the DNA sequence encoding SpCas9 plus sgRNA is approximately 4.2 kb, which is very close to the packaging limit of the widely used delivery system adeno‐associated virus (AAV) (about 4.7 kb),^24^ restricting its applications. Hence, studies aiming to overcome these shortcomings of SpCas9 nuclease have attracted attention, especially the investigations regarding alternative Cas9 orthologs that are condensed in size, with higher specificity and similar DNA editing capacity.

### 2.2 SaCas9

The *Staphylococcus aureus* Cas9 (SaCas9) system is another widely studied CRISPR‐Cas9 system.^20^ SaCas9 is 1053 aa in size (about 3.2 kb), which is much smaller than SpCas9, thus enabling the simultaneous carrying of the Cas9 and sgRNA coding sequences in a single AAV vector.^20^ A crystallographic study has shown that SaCas9 has a similar bi‐lobed structure to SpCas9, although they shared only 17% sequence identity (Figure 1A).^20^ SaCas9 recognizes distinct PAM sequence 5′ NNGRRT (Table 1).^20^ It is worth noting that while the pre‐requisite of a longer PAM could largely reduce the off‐target probability, it, however, reduces the number of potential targetable sites at the same time. Engineered variants of SaCas9 have been generated to recognize different PAM sequences such as 5′ NNNRRT, which provides opportunities to broaden the targeting range of CRISPR‐SaCas9.^25^

### 2.3 Other type II CRISPR‐Cas systems

As the earliest CRISPR‐Cas systems identified and applied for genome editing, the type II family keeps providing new choices of Cas effectors. Other than the above‐mentioned SpCas9 and SaCas9, representatives of type II CRISPR‐Cas systems also include *Campylobacter jejuni* Cas9 (CjCas9), *Streptococcus thermophilus* Cas9 (St1Cas9), *Neisseria meningitidis* Cas9 (NmCas9) and *Francisella novicida* Cas9 (FnCas9) (Table 1 and Figure 1A). CjCas9 (984 aa, about 3.0 kb) is the smallest Cas9 identified so far.^26^ Its PAM sequences are reminiscent of the long PAM sequence for SaCas9, but vary among different reports.^21^, ^26^ The condensed size of CjCas9 has enabled the packaging of its coding sequence, together with a sgRNA cassette and a marker gene, in an all‐in‐one AAV vector for genome editing.^26^ Remarkably, St1Cas9 (1122 aa, about 3.4 kb) and NmCas9 (1083 aa, about 3.2 kb) also have small sizes comparable to that of SaCas9 and show less stringent PAM requirements empirically (5′ NNAGAAW for St1Cas9 and 5′ NNNNGATT for NmCas9, respectively) (Table 1),^27^ which favours their application in genome editing.^28^, ^29^ Distinctly from most Cas9 orthologs among the type II families, FnCas9 does not resemble their bi‐lobed architecture while still contains the RuvC and HNH domains for nucleic acid cleavage.^30^ FnCas9 is 1629 aa in size (about 4.9 kb) with a 5′ NGG PAM, neither of which is an advantage over SpCas9 or SaCas9 for gene editing,^30^ but its E1369R/E1449H/R1556A mutant (RHA FnCas9, Table 1) can recognize the 5′ YG PAM, thus providing more target choices in the genome.^30^

## 3 TYPE V: THE CRISPR‐CAS12 SYSTEMS

Identification and characterization of Class 2 CRISPR‐Cas systems other than the type II systems have helped expand the CRISPR‐Cas arsenal for nucleic acid editing.^31^ Among them, Cpf1 (later renamed as Cas12a) was the earliest to be characterized,^32^ and subsequently, the C2c1 (Cas12b) and other type V (Cas12) systems were identified (Table 1 and Figure 1B).^33^, ^34^, ^35^, ^36^, ^37^ Although type V CRISPR‐Cas systems show great diversities, they still share some common characteristics that would distinguish them from the Cas9 systems. Firstly, the Cas12 nucleases possess one RuvC nuclease domain but no HNH domain, and they would recognize T‐rich PAM 5′ upstream to the target region on the non‐targeted strand, which is different from Cas9 systems which rely on G‐rich PAM at 3′ side of target sequences (Table 1 and Figure 1B).^32^ Secondly, Cas12 generates staggered DSBs distal to the PAM sequence, unlike Cas9, which generates a blunt end in the proximal site close to the PAM.^32^ The staggered DSBs created by Cas12 may support a unique targeting strategy for gene knock‐in via the non‐homologous end‐joining (NHEJ) mechanism.^32^ Despite these common properties, the Cas12 systems present vast structural and functional diversities. In the following paragraphs, members of the type V CRISPR‐Cas family that have been characterized will be introduced.

### 3.1 Cas12a (Cpf1)

Cas12a was the first functionally characterized type V CRISPR‐Cas system.^32^ The sizes of Cas12a family proteins vary from about 1200 aa (3.6 kb) to about 1500 aa (4.5 kb) (Table 1 and Figure 1).^32^ Cas12a proteins adopt a bi‐lobed architecture consisting of a REC lobe and a NUC lobe, which is reminiscent of Cas9,^38^ meanwhile they possess distinct characteristics making it worthwhile to develop new gene editing techniques using Cas12a. Cas12a proteins generally recognize 5′ TTN as the PAM sequence, except for *Acidaminococcus *sp. Cas12a (AsCas12a) that recognizes 5′ TTTN (Table 1).^32^, ^39^ Moreover, Cas12a proteins do not need tracrRNA for crRNA maturation; and Cas12a with crRNA alone can mediate robust DNA targeting and cleavage.^32^ While Zetsche et al showed that Cas12a contains the RuvC‐like endonuclease domain,^32^ the putative Nuc identified subsequently is not an active nuclease domain (Figure 1B).^39^ Upon binding of the target DNA, Cas12a‐crRNA complex induces a nick in each of the target DNA strands, yielding a sticky‐end‐like DNA DSB of 4 or 5 nucleotides (nt) overhang.^32^ In addition to the *cis‐*cleavage of the target DNA, Cas12a also triggers *trans*‐cleavage of non‐target DNAs.^40^ Several Cas12a orthologs, including AsCas12a, *Lachnospiraceae bacterium* Cas12a (LbCas12a) and *Francisella novicida* Cas12a (FnCas12a), have been found to have indiscriminate single‐stranded (ss) DNase activity upon binding to its target DNA sequence(s) (Table 1 and Figure 1).^41^ Although the exact mechanism has not been fully understood, studies have suggested that while the *cis‐*cleavage by Cas12a requires recognition of PAM and a specific target sequence, the non‐target DNA degradation through the *trans*‐cleavage occurs in a PAM‐ and sequence‐independent manner.^42^, ^43^ It is noteworthy that the *trans*‐cleavage activity by Cas12a has been re‐purposed for highly sensitive detection of specific nucleic acid sequence(s).^41^

### 3.2 Cas12b (C2c1)

The Cas12b proteins are another group of type V CRISPR‐Cas effectors that have DNA‐cleaving activity (Table 1 and Figure 1B).^34^ *Alicyclobacillus acidoterrestris* Cas12b (AacCas12b), an example of Cas12b effectors, is 1129 aa in size (about 3.4 kb), similar to that of SpCas9 and Cas12a.^44^ In general, Cas12b recognizes 5′ T‐rich PAM as Cas12a does, while different orthologs in the Cas12b family require different PAM sequences such as 5′ TTN for AacCas12b and 5′ ATTN for *Bacillus thermoamylovorans* Cas12b (BthCas12b) (Table 1 and Figure 1B).^34^ Unlike Cas12a, however, Cas12b requires both crRNA and tracrRNA to form an effector complex to proceed to the targeted DNA cleavage, which yields staggered DSBs with seven‐nucleotide overhangs.^33^, ^44^ Interestingly, structure analysis showed that Cas12b employs a distinct strategy for target DNA recognition and cleavage when compared to Cas12a and Cas9. AacCas12b, for example, binds to the sgRNAs as a binary complex and then to target DNAs as ternary complexes, which permits the capture and cleavage of both the target and non‐target DNA strands independently.^14^, ^44^ It is worth noting that the Cas12b system is highly sensitive to any single‐base mismatch within the 20 nt spacer region, which suggests high specificity of CRISPR‐Cas12b systems.^33^ However, it should also be noticed that some Cas12b effectors, such as AacCas12b, possess ssDNA‐cleaving activity similar to Cas12a.^41^

### 3.3 Other type V CRISPR‐Cas systems identified from metagenomic data

Recently, several other type V CRISPR‐Cas members besides Cas12a and Cas12b have also been identified from metagenomic data through bioinformatic pipelines (Table 1 and Figure 1B).^35^, ^36^, ^37^ While members of the Cas 12 systems all share the common characteristics including a single RuvC nuclease domain and T‐rich PAM (except for Cas12g), it should also be noted that they do present a great extent of functional and structural variety (Table 1 and Figure 1B). Cas12d (CasY) does not need a tracrRNA for normal functioning, which is similar to the cases of Cas12a. Cas12e(CasX), Cas12g, and Cas12h are of sizes less than 1000 aa, while Cas14a1 (belonging to subtype V‐F) has a miniature size of 529 aa. Cas12g1 cleaves single‐stranded RNA (ssRNA) and ssDNA rather than dsDNA, and it does not require PAM sequences for target sequence recognition (Table 1).^35^, ^36^, ^37^ Many of these Cas12 proteins exhibit collateral ssDNA‐cleaving activity after the effector complexes bind to their target sequence(s).^35^, ^37^

## 4 TYPE VI: THE CRISPR‐CAS13 SYSTEMS

Up to now, reported members of the type VI CRISPR‐Cas family include Cas13a, Cas13b, Cas13c and Cas13d (Table 1 and Figure 1C). Distinct from Cas9 and Cas12, the Cas13 proteins possess unique properties to cleave ssRNA rather than DNA.^45^ The subtypes of the Cas13 systems have their unique features while sharing some common characteristics. There is no DNA catalytic domain in Cas13 proteins; instead, researchers identified two conserved higher eukaryotes and prokaryotes nucleotide‐binding (HEPN) domains, each containing an RNA cleavage site (Figure 1C).^45^

Members of the CRISPR‐Cas13 system work as dual‐component systems, in which a crRNA forms a complex with the Cas13 protein without involving any tracrRNA.^46^ The flanking sequence(s) of protospacers, termed as “protospacer‐flanking site” (PFS) and comparable to the “PAM” for Cas9 and Cas12, is essential for the RNA‐targeting process (Table 1).^45^

Another distinctive feature of the Cas13 systems is the collateral cleaving activity towards non‐targeted, unspecific RNAs in the reaction environment. Upon binding with the targeted RNA, the catalytic pocket formed by the two HEPN domains is activated and can cleave exposed RNA indiscriminately in the solution, including endogenous RNAs of housekeeping genes.^46^ This promiscuous RNase activity may protect bacteria from virus spread via infection‐triggered cell death and dormancy induction.^45^ Several subtypes of CRISPR‐Cas13 systems have been introduced as potential tools for RNA editing. Among them, the structures and activities of Cas13a, Cas13b and Cas13d have been studied (Table 1 and Figure 1C).^45^, ^47^, ^48^

### 4.1 Cas13a (C2c2)

The CRISPR‐Cas13a (C2c2) is the most thoroughly studied system in the Cas13 family, represented by two members *Leptotrichia shahii* Cas13a (LshCas13a, 1389 aa in size, ~4.2 kb in length) and *Leptotrichia wadei* Cas13a (LwaCas13a, 1152 aa in size, ~3.5 kb in length) (Table 1 and Figure 1C).^45^, ^49^ LshCas13a requires the 3′ non‐G PFS downstream to the target sequence, which is less stringent than the PAM required by Cas9 and Cas12.^45^ LshCas13a‐mediated target RNA cleavage is sensitive to double or consecutive mismatches in the central region of the spacer, supporting the existence of a central “seed” region.^45^ Upon activation by target RNA binding, the HEPN catalytic sites of Cas13a are exposed to the surface far from where the guide‐target RNA duplex is held inside the NUC lobe.^50^ This phenomenon suggests that only long RNAs can be cleaved in *cis*, and short RNAs may be cleaved in *trans*.^50^ Studies have shown that HEPN mutations can result in a catalytically inactive, programmable, RNA‐guided RNA binding protein, which could be applied for tracking specific transcripts in living cells^45^, ^49^ or RNA base editing.^51^

### 4.2 Cas13b

The Cas13b system is another member of type VI CRISPR‐Cas13 systems characterized after Cas13a (Table 1 and Figure 1C).^47^ The Cas13b loci always encode a large effector of about 1100 aa in size (~3.3 kb) and a small accessory protein of about 200 aa.^47^ Cas13b systems can be categorized into two variant systems denoted VI‐B1 (*Bergeyella zoohelcum* Cas13b, BzCas13b, in which its accessory protein is referred to as Csx27) and VI‐B2 (*Prevotella buccae* Cas13b, PbCas13b, in which its accessory protein is referred to as Csx28).^47^ Csx27 and Csx28 exhibit antagonistic roles, that is Csx27 represses Cas13b activity, and Csx28 stimulates Cas13b activity.^47^ The PFS determination shows that Cas13b requires double PFS: 5′ D at the 5′ side and 5′ NAN/NNA at 3′ the side (Table 1).^47^ The possibilities of a controllable Cas13b activity by Csx27 or Csx28 and an improved targeting specificity by involving double PFS provide new opportunities to expand the CRISPR toolbox further.

### 4.3 Cas13d

Cas13d is a new RNA‐targeting effector whose crystal structure has recently been defined (Table 1 and Figure 1C).^52^ Similar to other Cas13, it has two HEPN domains responsible for pre‐crRNA processing (Figure 1C).^53^ Meanwhile, Cas13d possesses appealing characteristics for RNA editing. Its small size (less than 1000 aa, 3 kb) favours the delivery to designated organ systems via AAV, and the lack of PFS requirement makes it less restrictive in selecting target sequences for RNA editing.^48^

## 5 HIGH‐EFFICIENCY GENOME EDITING AND NEW APPLICATIONS ENABLED BY CATALYTICALLY ACTIVE CAS9 AND CAS12

The unprecedented high efficiency, site‐specific targeting and ease of programming have made the first application of CRISPR‐Cas9 in genome editing a hallmark in biomedical research. The subsequent synergy between the extensive examination of potentials and limitations regarding diverse CRISPR‐Cas systems in genome editing, and the development of various targeting strategies by exploiting different DNA repair mechanisms, has prompted vast new applications.

### 5.1 Introducing insertions or deletions through targeted DNA cleavage followed by NHEJ repair

Cas9 and Cas12 cleave dsDNA and generate DSBs with blunt and staggered ends, respectively. The subsequent repair via the highly active but error‐prone NHEJ mechanism can lead to the efficient introduction of small insertions/deletions (indels) at the target sites, which disrupts the translational reading frame of a coding sequence or the binding sites of trans‐acting factors in promoters or enhancers. The first evidence for successful genome editing mediated by CRISPR‐Cas9 in mammalian cells was the detection of indels specific to the pre‐selected target sites after Cas9 cleavage.^5^, ^6^ Soon this strategy was applied in mouse zygotes and achieved the one‐step generation of *Tet1/Tet2* double knockout mouse line.^54^ A myriad of genomic modifications enabled by adopting Cas9 cleavage followed by NHEJ repair has been reported, such as fragment deletion, reversion and chromosomal translocation. Meanwhile, tremendous efforts have been made to direct genomic cleavage using different Cas, among which, the unique capability of Cas12 in the generation of staggered ends has brought improvement to the accuracy of ligation between broken ends via the NHEJ repair.^32^

Recently, the NHEJ repair mechanism has also been exploited to capture large foreign DNA at Cas9‐induced DSBs, thus establishing a new homology‐independent knock‐in approach.^55^ With the aid of a promoterless reporter system targeting at the universally expressed *GAPDH*, He et al have carried out a side‐by‐side comparison between homology‐directed repair(HDR)‐ and NHEJ‐mediated knock‐in. They found that the NHEJ pathway mediated homology‐independent knock‐in at previously unattainable high efficiencies, which was superior to commonly used HDR methods in all human cell lines examined.^56^ Subsequently, Zhang et al have applied this strategy to trace and enrich gene disruption in hyperploid somatic cell lines^57^; and Suzuki et al^58^ applied this strategy for in vivo transgene integration, which has directed its application for genome editing‐based therapy. It is noteworthy that NHEJ‐mediated knock‐in introduces desired modifications as well as reversely oriented insertions, and it yields indels at integration junctions,^56^, ^58^ which should be taken into consideration to prevent unwanted outcome in subsequent applications.

### 5.2 Targeted sequence replacement via CRISPR‐induced HDR repair

Targeted knock‐in of foreign DNA into a selected genomic locus was first established through homologous recombination without cleavage in the genome. Despite the low efficacy and cumbersome procedures, this traditional HDR‐based DNA replacement strategy has achieved far‐reaching success in generating numerous genetically modified mouse lines.^59^ Studies have found that the site‐specific DNA cleavage could significantly increase the efficiency of HDR‐based knock‐in at the nearby region by up to ~1000‐folds,^60^ which widely prompts the application of CRISPR‐HDR‐based methods to introduce various genomic modifications through sequence replacement. The homologous templates have been provided in various forms, ranging from a small single‐stranded oligonucleotide (ssODN) with a size of around 90‐120 nt,^61^ large circular (plasmids) or linear dsDNA,^61^, ^62^ to ssDNA delivered via AAV vectors.^63^ Through CRISPR‐induced HDR repair, gene function analysis and disease modelling/correction have become feasible via introduction and correction of specific point mutations,^64^ or targeted insertions of desired sequences ranging from a few nucleotides to large DNA fragments up to 7.5 kb.^65^ Importantly, with the assistance of CRISPR‐Cas9 systems, HDR‐based gene targeting has become possible in previously non‐permeable cell models, such as human embryonic stem cells (ESCs) and induced pluripotent stem cells (iPSCs).^41^, ^66^, ^67^ Despite the fact that the HDR mechanism mediates precise knock‐in of desired sequences, recent studies showed that unwanted insertions of donor templates and high frequency of indels at target sites were introduced through NHEJ repair, which might not be avoidable.^61^, ^62^, ^68^ Hence, further investigations into the functional impact of these unwanted modifications would be of interest.

### 5.3 Advanced technologies for transgenesis

The programmable targeted genome editing brought about by the revolutionary CRISPR‐Cas9 systems has enabled genetic modifications that were previously impossible, such as those in lower vertebrates and large mammals.^55^, ^69^ The CRISPR‐based technology has widely revolutionized the field of transgenesis, and now it is possible to generate genetically modified animal models in almost any species. In line with this development, various delivery methods have been established. For small organisms, the Cas9, sgRNA and donor templates can be easily delivered in forms of plasmids, mRNAs or in vitro assembled ribonuclear protein complexes (RNPs) through direct injection into the gonads of, for example, the *C elegans*,^70^ or into the zygotes and pre‐blastoderm embryos of mice^61^ and *Drosophila*.^71^ Compared to the conventional transgenic technologies that involve a series of labour‐intensive procedures and often take more than a year to produce genetically modified mice,^72^ the CRISPR‐Cas9 tools have dramatically speeded up this process. For instance, Jaenisch's group has reported a successful one‐step generation of mouse lines carrying targeted insertions of three different tags and fluorescence reporters at *Nanog, Sox2* and *Oct4* loci in a pre‐designed manner.^61^

The CRISPR‐Cas9‐based genome editing via direct zygotic injection has also overcome many previous hurdles and enabled targeted genomic modifications in large mammals, including non‐human primates,^69^, ^73^, ^74^ pigs^75^ and cows.^76^ For example, Sha and his colleges have demonstrated precise editing of the *Ppar‐g* and *Rag1* genes simultaneously through the zygote injection of the Cas9 mRNA and five different sgRNAs in Cynomolgus monkeys.^69^ Studies have also disrupted the *Dax1* gene in monkeys to recapitulate human X‐linked adrenal hypoplasia congenita or mutated the dystrophin gene to induce Duchenne muscular dystrophy, providing invaluable models for disease investigations and therapeutic development.^73^, ^74^ Collectively, the CRISPR‐Cas‐based genome editing strategies have greatly extended the capabilities of transgenic technologies to the previously unimaginable areas by including extra‐complicated genetic modifications and dealing with new species, presenting high potentials in agricultural and pharmaceutical applications.

### 5.4 Genome editing for disease therapy

Other than introducing genetic modifications that can be passed on across generations, CRISPR‐Cas systems have also enabled the previously impossible somatic genome editing owing to the high editing efficiency permitted. By coupling with the AAV system, which is a clinically potent and safe vector for in vivo gene delivery,^77^ CRISPR‐based genome editing has been successfully achieved in living animals. The successful in vivo editing by AAV‐CRISPR systems was first achieved as the introduction of small indels to disrupt endogenous genes such as *Pcsk9* and *Mecp2*.^78^, ^79^ Subsequently, the deletion of *Dmd* exon 23 with a pathogenic mutation,^80^, ^81^ and the mutation correction in *Otc* or *F9* genes through HDR‐mediated sequence replacement,^82^, ^83^ have been shown to reverse related disease symptoms successfully. More recently, a study reported knock‐in of the *hF9* gene using an AAV‐delivered CRISPR system and confirmed the reversal of haemophilia B symptoms in a mouse model.^84^

Applying CRISPR‐Cas9 under ex vivo conditions may be technically less challenging but equally valuable for disease therapy. For instance, ex vivo genome editing in hematopoietic stem cells (HSCs) could potentially provide a cure for patients carrying sickle cell disease (SCD) and β‐thalassaemia,^85^ and targeted insertions via CRISPR strategies may provide a valuable alternative to T‐cell engineering for cancer immunotherapy.^86^ A proof‐of‐concept study by Dewitt et al^87^ has reported an efficient correction of the E6V mutation at sickle alleles in patients' HSCs, through CRISPR‐HDR‐based replacement with the assistance of oligonucleotide donors. Using AAV‐delivered Cpf1, Dai et al^86^ built stable CAR‐T cells with immune‐checkpoint knockout (KIKO CAR‐T cells) via the HDR‐based strategy in one step.

Recent studies have also attempted treating infectious diseases using CRISPR‐Cas9 technologies. Targeted mutations at the conserved sites in long terminal repeats (LTR) or U3 region in the proviral DNA of human immunodeficiency virus‐1 (HIV‐1) have shown great potentials as new anti‐HIV therapies.^88^ Similar strategies have also been employed to combat other virus‐originated infectious diseases, such as hepatitis B virus (HBV)^89^ and hepatitis C virus (HCV).^90^

### 5.5 Genome‐wide functional screening through CRISPR‐Cas9 technologies

Catalytically active Cas proteins, mainly the SpCas9, have been applied with libraries of sgRNAs to provide a new functional genomics approach to cater to the needs of genome‐wide knockout screenings.^91^ In pioneer studies, Shalem et al constructed an sgRNA library consisting of 64,751 unique guide sequences targeting 5′ exons of 18,080 human genes and identified new genes involved in drug resistance to a therapeutic RAF inhibitor, vemurafenib.^92^ Independently, Wang et al constructed another sgRNA library containing 73,000 sgRNAs that targeted coding exons and conducted screening using a promyelocytic leukaemia (PML) cell line HL60, which identified *TOP2A* and *CDK6* as new genes responsible for the drug resistance to purine analogue 6‐thioguanine.^93^ Subsequent screenings also discovered other genes critical to cancer development and treatment,^94^, ^95^, ^96^ such as *BCR* and *ABL*, which could be potentially regarded as new therapeutic targets in leukaemia and colorectal carcinoma.^94^ Moreover, the CRISPR‐based knockout screenings have also been carried out in vivo to probe the complex interaction between cancer cells and the microenvironments to identify the corresponding oncogenes,^97^ tumour suppressors,^96^ and immune regulators.^98^

Applications of CRISPR‐based screenings are being expanded rapidly. Up to now, sgRNA libraries have been constructed for both human^92^, ^93^, ^97^ and murine models,^99^, ^100^ with target sites not only in the coding but also in the non‐coding regions.^101^ Future research might solve the systemic limitations such as restrictions due to PAM requirements, challenges for working with hyperploid cell models to generate loss‐of‐function phenotypes and compensatory effects introduced by survival stress when an essential gene is knocked out.^57^

## 6 NOVEL TECHNOLOGIES BASED ON THE SITE‐SPECIFIC GUIDANCE BY CRISPR‐CAS9 AND ORTHOLOGS

The principle of RNA‐guided DNA targeting by CRISPR‐Cas9 systems has been expanded to different usages by fusing Cas9 with various functional proteins or domains. A catalytically inactive version of Cas9 (catalytically dead Cas9, or dCas9) can be produced through mutagenesis (D10A and H841A) of the two nuclease domains so that the dCas9 together with sgRNA can work as a sequence‐specific guide which may carry a functional protein or domain to desired loci in the genome without creating DNA breaks.^102^ Examples of the attached functional proteins range from deaminases^103^ and transcription factors (activators and repressors)^104^ to labelling molecules,^105^ which will be discussed in this part.

### 6.1 Base editing by fusing Cas9 with different deaminases

Cytidine deaminases such as APOBEC1 and AID catalyse the conversion of cytidine to thymidine (C → T) and, when fused with a dCas9 or nickase Cas9 (D10A), become a novel tool to mediate C•G to T•A substitutions within targeted sequences, which is now known as the base editor (BE).^103^ As no enzymes are known to deaminate adenine in DNA, an RNA adenosine deaminase is fused to mutant Cas9 to realize specific A•T to G•C substitution in DNA.^106^ Both types of BEs can catalyse base substitution in the genome without DNA cleavage, which can be applied under either in vitro or in vivo conditions. Since the first demonstration by Komor et al,^103^ base editing systems, primarily the third‐generation BE (BE3), have been applied in a wide range of cell types, including various cell types from human and mouse.^103^, ^107^ Successful base editing has also been achieved in living animals such as mice^108^ and in zygotes to generate transgenic strains in mice^109^, ^110^ and zebrafish.^111^

As most genetic diseases are caused by point mutations, BE‐mediated site‐specific base substitution holds the potentials for therapeutic applications. Studies by Kim et al and Komor et al have reported various base editing systems by employing Cas9 variants with different PAM sequence specificities, including SpCas9 (5′ NGG), SaCas9 (5′ NNGRRT) and artificially evolved Cas9, which are found to be promising for correction of more than 2,000 potential pathogenic point mutations.^107^, ^112^

The BE systems have also been applied for genome‐wide knockout or mutagenesis screenings. The CRISPR‐STOP^113^ and iSTOP^114^ systems have employed BE3 to introduce stop codons at arginine, glutamine and tryptophan residues, efficiently disrupting coding sequences and generating targeted truncations. Meanwhile, the CRISPR‐X and targeted AID‐mediated mutagenesis (TAM) systems were developed by fusing dCas9 with AID carrying P182X mutation (AIDx),^115^, ^116^ which could convert cytidines (or guanines) into the other three bases, generating a vast repertoire of variants in a target protein. These technologies have been applied for high‐throughput screenings of functional variants or rapid evolution of human antibodies through targeted mutagenesis.^117^ Limitations associated with the BE‐based strategies have been investigated recently, and Grünewald et al^118^ showed that the DNA‐base editing could cause transcriptome‐wide deamination of RNA cytosines and potentially result in extensive off‐target RNA base editing in human cells. Nevertheless, further investigations are necessary to explore the full potentials as well as to overcome the limitations of these newly emerged base editing tools.

### 6.2 Targeted transcriptional and epigenetic regulation via Cas9‐guided activators or repressors

Besides gene editing, the CRISPR‐Cas9 systems have also been employed as RNA‐guided platforms to mediate transcriptional or epigenetic regulations with respect to a targeted gene. Catalytically inactive Sp dCas9 has been fused with different transcriptional activators (VP64, p65AD, SunTag or VPR) or repressors (KRAB) to generate synthetic transcription factors.^104^, ^119^, ^120^, ^121^ Studies found that the up‐regulation of targeted gene expression could be augmented by fusing dCas9 with VP64 in the form of multiple repeats (up to 10 copies), and targeting proximal promoter regions with multiple sgRNAs could largely activate endogenously non‐expressed genes.^119^ In contrast, Thakore *et al* have shown that the fusion of dCas9 with KRAB repressor could silence gene expression through H3K9 trimethylation.^120^ In 2013, Qi et al^102^ further outstretched this strategy by applying dCas9‐KRAB with sgRNA libraries and thus established a new method for genome‐wide screenings, termed CRISPR interference (CRISPRi). Subsequently, studies employed the dCas9‐activator for a genome‐wide CRISPR activation (CRISPRa) screening. Gilbert et al^121^ identified genes that are responsible for the growth of K562 cells or sensitivity towards a toxin (CTx‐DTA), while Bester et al^122^ investigated the possible role of long non‐coding RNAs (lncRNAs) in drug resistance.

In addition to transcriptional regulation, dCas9 systems have also been re‐purposed for epigenetic remodelling. Maehr's group fused the Nm dCas9 to a histone demethylase LSD1 and visualized efficient disruption effects of Nm dCas9‐LSD1 against targeted enhancers.^123^ Meanwhile, Gersbach's group fused dCas9 to the catalytic core of human acetyltransferase p300, which mediated acetylation of histone H3 lysine 27 at the target site, leading to robust transcriptional activation of targeted genes.^124^

### 6.3 CRISPR‐enabled high‐resolution genomic imaging in live cells

The superior capability of CRISPR‐Cas9 for RNA‐guided DNA binding is compelling for targeting and visualization of specific genomic structures and dynamics in cells and tissues. Chen et al^105^ firstly recorded the dynamics of repetitive genomic loci using EGFP‐tagged dCas9 guided by a single sgRNA throughout the cell cycle in living cells, followed by tracking of some non‐repetitive loci by co‐delivering an array of sgRNAs tiling along the target locus. Subsequently, the application of Cas9‐based imaging has been broadened by employing different labelling strategies and coupling with a variety of other tags. Tanenbaum et al have fused dCas9 with the SunTag peptide array to recruit 24 copies of GFP‐fusion proteins to the targeted sequences for amplification of the fluorescence signals and achieved long‐term tracing of single protein molecules.^125^ Ma et al^126^ developed a series of multicolour genomic labelling systems by engaging three dCas9 orthologs (Sp dCas9, Nm dCas9 and St1 dCas9) fused with different fluorescent proteins, or combining single Sp dCas9 with synthetic guide RNA scaffolds that bind sets of fluorescent proteins. The later technology, termed CRISPRainbow, has enabled simultaneous tracking of up to six chromosomal loci in a living cell.^126^ In other studies, Knight et al^127^ fused dCas9 to Halo‐Tag to visualize the diffusion and chromatin navigation process of Cas9 complex in living cells through single‐particle tracking; while David et al have extended the utility of the dCas9‐based imaging system and achieved endogenous RNA tracking in living cells as well.^128^

## 7 PROSPECTS

Continuous discoveries of new CRISPR‐Cas systems and engineering of existing ones are rapidly expanding the molecular toolbox for nucleic acid manipulations. On the one hand, bioinformatics has contributed significantly to discovering more CRISPR‐Cas systems from nature through genome‐ or metagenome‐wide searching and screening, yielding the majority of type V and type VI CRISPR‐Cas systems.^35^, ^36^, ^37^ On the other hand, a particular type of Cas proteins (*eg* SpCas9) can be modified through structural engineering, either in ways that resemble directed evolution for improved PAM specificities^129^, ^130^ or by fusing with a plethora of proteins for extended functions,^131^ catering for different research needs. The increasing numbers and diversity of available Cas orthologs and their artificial mutants have much overcome the natural limitations associated with individual effectors, while the fused proteins endow Cas protein abilities beyond nucleic acid editing.

Off‐target editing at sequences similar to selected target sites has been observed with some commonly used Cas proteins (*eg* SpCas9 and SaCas9), which raises a critical concern to CRISPR‐Cas technologies and it is especially undesirable for therapeutic applications.^22^ Aiming at a thorough and unbiased detection of off‐target events, multiple experimental methods have been established to capture the genomic landscape of CRISPR–Cas9 cleavages through high‐throughput sequencing technology, which include GUIDE‐seq, CIRCLE‐seq and SITE‐Seq.^132^, ^133^, ^134^ While these technologies showed high sensitivity in detecting off‐target events, some limitations were also noticed, including restrictions to cell models and high rates of false positives introduced by complicated experimental procedures.^135^ At the same time, strategies have been adopted for enhancement of the specificity of CRISPR‐Cas tools through protein engineering, sgRNA modifications and selection of suitable delivery methods.^136^ For example, the enhanced specificity SpCas9 (eSpCas9)^137^ and high‐fidelity variant number 1 of SpCas9 (SpCas9‐HF1)^138^ are improved versions of SpCas9 proteins, of which mutations have been induced for reduction of electrostatic^137^ and energetic^138^ interaction affinities with targeted DNA. More recently, Tan et al also reported a high‐fidelity SaCas9 variant, which showed improved specificity and unchanged on‐target editing efficiencies in the genome‐wide analyses.^139^

Furthermore, owing to the needs of temporal control over gene activities in some study fields, inducible CRISPR‐Cas systems have been attempted through chemical induction and optogenetics,^131^ accomplishing significant controllability by coupling of chemical‐ or light‐sensitive molecules with dCas9.^131^ In summary, the CRISPR‐Cas technologies and its associated applications are expeditiously evolving and, thus, enabling numerous novel applications whose future development may go beyond the scopes that we could currently foresee.

## CONFLICT OF INTEREST

The authors declare no competing financial interests.

## AUTHOR CONTRIBUTIONS

J.W. drew the figure and the table; J.W., C.Z. and B.F. wrote the paper. All authors have read and approved the final manuscript.

## Data Availability

The data that support the findings of this study are available from the corresponding author upon reasonable request.
